# Supplementary figures and images for: Isolation and Characterization of Copper- and Zinc- Binding Metallothioneins from the Marine Alga Ulva compressa (Chlorophyta)
Source: Int J Mol Sci. 2019 Dec 25;21(1):153. doi: 10.3390/ijms21010153 (PMC6981760; doi:10.3390/ijms21010153)

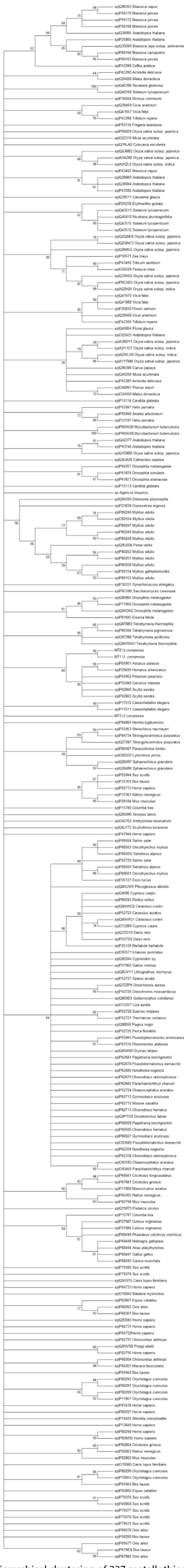

**Fig S1.** Hierarchical clustering of 237 metallothionein sequences, including UcMTs

Supplement: Supplementary file 1 [file ijms-21-00153-s001.pdf]
